# Risk Factors of Eye Complications in Patients Treated in the Intensive Care Unit

**DOI:** 10.3390/ijerph182111178

**Published:** 2021-10-25

**Authors:** Lucyna Płaszewska-Żywko, Aurelia Sega, Agnieszka Bukowa, Katarzyna Wojnar-Gruszka, Marcelina Podstawa, Maria Kózka

**Affiliations:** 1 Department of Clinical Nursing, Faculty of Health Sciences, Jagiellonian University Medical College, 31-126 Krakow, Poland; lucyna.plaszewska-zywko@uj.edu.pl (L.P.-Ż.); k.wojnar-gruszka@uj.edu.pl (K.W.-G.); marcelina.podstawa@uj.edu.pl (M.P.); maria.kozka@uj.edu.pl (M.K.); 2 Department of Anesthesiology and Intensive Therapy, University Hospital in Krakow, 31-688 Krakow, Poland; abukowa@su.krakow.pl

**Keywords:** eye complications, critically ill patient, eye care, intensive care unit (ICU)

## Abstract

In critically ill patients, normal eye protection mechanisms, such as tear production, blinking, and keeping the eye closed, are impaired. In addition, many other factors related to patients’ severe condition and treatment contribute to ocular surface disease. Reducing risk factors and proper eye care can have a significant impact on incidences of eye complications and patient quality of life after discharge from the intensive care unit (ICU). The aim of the study was to determine risk factors for ocular complication, especially those related to nursing care. The study was conducted in the ICU of a university hospital. Methods for estimating and analyzing medical records were used. The patient’s evaluation sheet covering 12 categories of risk factors for eye complications was worked out. The study group included 76 patients (34 patients with injuries and 42 without injuries). The Shapiro–Wilk test, the Spearman’s rank correlation test, the Mann–Whitney U test and the Friedman’s ANOVA test were used. The level of significance was set at α = 0.05. The most important risk factors for eye complications in the study group were: lagophthalmos (*p* < 0.001), sedation (*p <* 0.01), use of some cardiological drugs and antibiotics (*p* < 0.01), mechanical ventilation (*p* < 0.05), use of an open suctioning system (*p* < 0.01), presence of injuries (*p* < 0.01) including craniofacial trauma (*p <* 0.001), high level of care intensity (*p* < 0.01), failure to follow eye care protocol (*p <* 0.001), length of hospitalization at the ICU (*p <* 0.001), and the frequency of ophthalmological consultations (*p <* 0.001). There was no correlation between the incidence of these complications and the age and gender of the patients. The exposure of critically ill patients to eye complications was high. It is necessary to disseminate protocols and guidelines for eye care in ICU patients to reduce the risk factors.

## 1. Introduction

Intensive care units (ICU) treat patients in life-threatening conditions who require the comprehensive care of an interdisciplinary team. During hospitalization, the medical staff mainly focuses on securing basic vital functions, controlling life-threatening disorders, and stabilizing the patient’s condition. Less pressing problems, including ocular complications, are sometimes overlooked by medical professionals [1,2].

Eye complications are common among ICU patients. Signs of ocular surface disease are found in 20–42% [3], and even up to 60% of ICU patients [4], with exposure to keratopathy in 37–57% of sedated and intubated patients [5,6]. Frequently identified eye diseases include superficial and direct injuries of the cornea—most often a superficial corneal abrasion (scratch), chemosis, which is conjunctival swelling, and microbial conjunctivitis and keratitis [3]. Eyelids not closing (lagophthalmos), which is a frequent cause of eye surface damage, occurs in 17–75% of ICU patients [4,7]. These complications may lead to irreversible pathological changes, blindness, disability, and deterioration in the quality of life of patients after ICU discharge [8,9]. Eye complications usually occur between the 2nd and 7th days of stay in the ICU [10,11].

The risk of ocular complications increases in persons with general injuries, craniofacial injuries (especially in the eye sockets [5]), in unconscious patients with severe conditions [3], and in respiratory system infections and ventilator-associated pneumonia (VAP). Another risk factor is the use of mechanical ventilation including Positive End-Expiratory Pressure (PEEP) and Continuous Positive Airway Pressure (CPAP), oxygen masks, and prone position [2,12,13,14,15]. Patients treated with sedatives, tranquilizers, and neuromuscular blockers are also at greater risk of developing eye complications [2,16].

Comprehensive daily care for intubated, mechanically ventilated patients is a routine nursing practice at the ICU. Eye care procedures vary widely between departments in terms of how often and how eyes are cared for. A review of the literature does not indicate clearly which eye care method is most effective. There is also a lack of unified eye care procedures based on scientific evidence [7,17,18]. ICU eye care protocols are not always implemented, and documentation of care in this area is often very limited. Nevertheless, some studies have shown that strictly adhering to these protocols contribute to the reduction of eye complications [5,7]. In Poland, in 2018, recommendations were developed based on the Evidence-Based Practice (EBP) guidelines [4,19] by the Working Group for the Practices of the Polish Society of Anesthetic and Intensive Care Nurses [20]. However, the practical application of these orders is far from optimal. The aim of the study was therefore to determine the risk factors for eye complications in ICU patients, especially those related to nursing care.

## 2. Material and Methods

The prospective research was conducted among patients treated in the ICU of a university hospital from February to April of 2019, after obtaining the consent of the hospital management and following the principles of the Helsinki Declaration. The study included patients with multi-organ trauma and isolated head injury (group 1), and patients without injuries, with circulatory disorders after a stroke and non-traumatic cerebral hemorrhage (group 2). During the study period, 163 patients were hospitalized in the ICU, of which 46 did not meet the eligibility criteria, 12 died on the first day of stay before the start of the study, and 23 died during the study before developing an eye complication. Data on 6 patients were rejected due to incomplete information in the documentation. Finally, the data about the remaining 76 patients (28 women and 48 men) aged 19–89 (mean 48 ± 17.9) were analyzed. Patients with injuries accounted for 44.7% (*n* = 34), and non-traumatic patients included 55.3% (*n* = 42).

The method of assessing and analyzing medical records (Therapeutic Intervention Scoring System—TISS observation cards, medical history, and nursing reports) was used. The patients were observed from the 1st to the 7th day of hospitalization. For this study, an original patient evaluation sheet was created. The risk factors for eye complications were assigned point values (from 1 to 6 points) in 12 categories: eye care (compliance with the protocol), use of mechanical ventilation, bronchial tree suction system, the intensity level of nursing care according to the Ministry of Health regulations (I—lowest, II—intermediate, III—highest level of care intensity) [21], use of sedation, use of other medications affecting the condition of the eyes, the presence of craniofacial and orbital trauma, the frequency of ophthalmic consultations, taking bacterial cultures, the presence of systemic bacterial infection (with particular attention to infections in the respiratory tract), spontaneous closing of the eyes, and the presence of accompanying eye diseases. Any of the following methods were qualified as proper eye care: rinsing with 0.9% NaCl or distilled water, using moisturizing drops and ointments, applying moisture chambers in patients with lagophthalmos, and antibiotic ointments in patients with conjunctivitis and keratitis. The respondents could obtain from 12 to 35 points in this research tool. The higher the score, the greater the risk of complications. Ocular complications were assessed according to the following criteria: no eye symptoms—1 point (pt.), conjunctival redness— 2 pts., broken vessels in the eye—3 pts., conjunctival hemorrhage—4 pts., conjunctival edema—5 pts., and purulent discharge in the eye—6 pts. The points from the whole period of observation were then summed up for analysis.

### Statistical Analysis Methods

The analysis was performed using the SPSS version 23 statistical program. In order to select appropriate methods of statistical analysis, the equivalence of the analyzed groups and the compliance of the distribution of variables with the normal distribution were checked using the Shapiro–Wilk test. The level of significance was set at α = 0.05. Since the variables did not conform to the normal distribution, the Spearman’s rank correlation test, the Mann–Whitney U test and the Friedman’s ANOVA test were used.

## 3. Results

**Figure 1:**
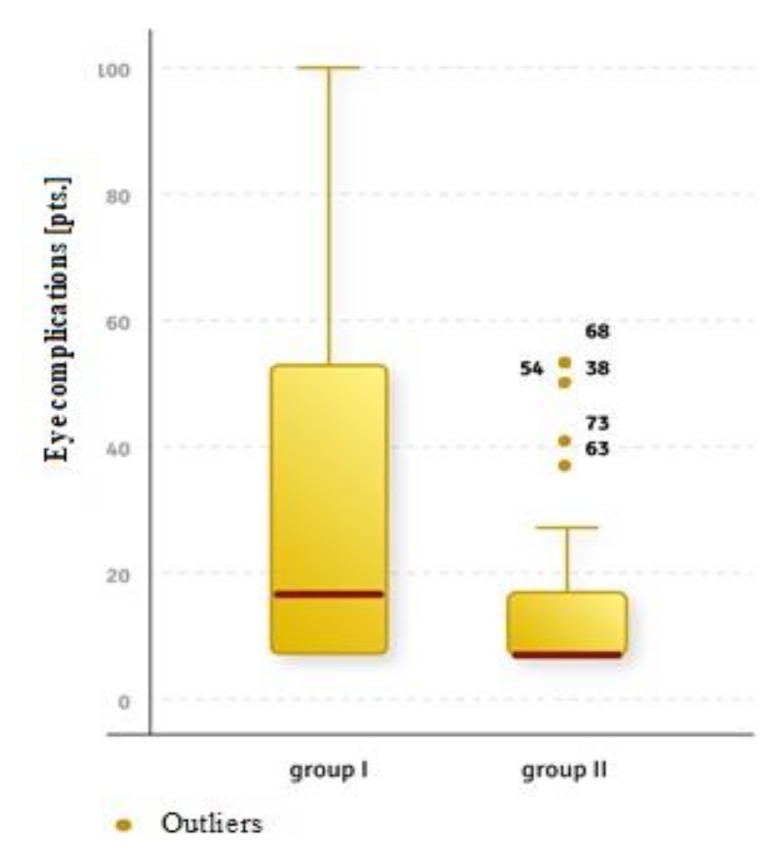
Comparison of the occurrence of ocular complications in patients with injuries (group 1) to those without injuries (group 2).

The study showed a statistically significant correlation between the incidence of eye complications and the medical diagnosis (U = 468.5, Z = −2.667, *p <* 0.01). A higher risk of ocular complications was observed in patients with injuries, meaning group 1 (Me = 16.5), rather than in patients without injuries in group 2 (Me = 7) (Figure 1). Moreover, a statistically significant relationship was demonstrated between the number of craniofacial and eye socket injuries and the frequency of eye complications (rho = 0.640, *p <* 0.001). The more craniofacial and orbital injuries there were, the greater the risk of developing these complications.

In the classification of the intensity of care for hospitalized patients (I–III) it was found that patients requiring more intensive nursing care had a higher risk of developing eye complications (rho = 0.309, *p <* 0.01).

Among patients who breathed independently, the incidence of eye complications were lower as compared to the mechanically ventilated group (rho = 0.250, *p <* 0.05). There was a higher risk of ocular complications in the sedated group as compared to the non-sedated group (rho = 0.373, *p <* 0.01). Moreover, more ocular complications were observed in patients who received drugs such as amiodarone, cardiac glycosides, anticoagulants, beta-blockers, penicillin, rifampicin, and metronidazole (rho = 0.424, *p* <0.001). The risk of complications was much higher in the group with lagophthalmos than in those who could close their eyelid and blink (rho = 0.659, *p <* 0.001).

**Figure 2:**
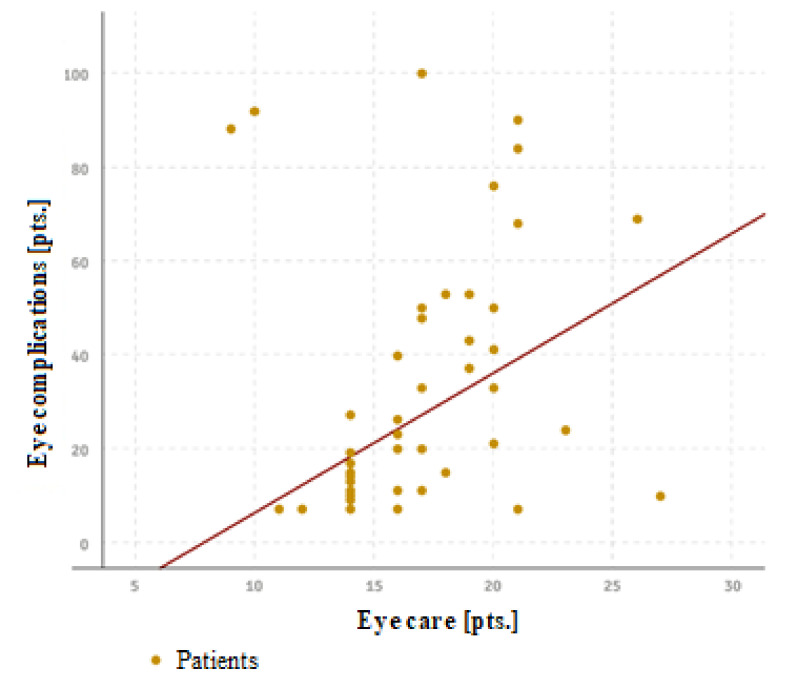
Relationship between the use of recommended eye care procedures and the occurrence of eye complications.

The studies also showed that, in patients in whom lack of the recommended eye care methods was observed, the frequency of complications increased significantly (rho = 0.590, *p <* 0.001), as seen in Figure 2.

A much higher frequency of ocular complications was observed in the group of patients under an open bronchial tree suction system than in patients using a closed suction system (rho = 0.389, *p <* 0.01).

The risk of ocular complications also depended on the frequency of ophthalmic consultations. The greater the frequency of these consultations, the lower the risk of complications (rho = −0.539, *p <* 0.001). Ocular complication risk also depended on the length of stay of the studied patients in the ICU (*p <* 0.001). To check the differences on individual days, a post hoc analysis was performed. There were no significant differences between the occurrence of complications from days 1–4, but an increase in the frequency of complications was observed on day 4, and the highest intensity was on day 7. There were statistically significant differences between the frequency and severity of complications on days 5, 6, and 7 than those on days 1, 2, 3, and 4 (*p <* 0.001, Table 1).

Statistical analysis showed no significant relationship between patient age (rho = −0.139, *p* > 0.05) and gender (U = 655.0, Z = −0.190, *p* > 0.05) and the risk of eye complications (Table 2).

## 4. Discussion

Incidences of ocular surface disease and other ophthalmic complications in ICU patients are high, ranging from 20% to 60% of cases, depending on the diagnosis criteria, the number of hospitalization days, and other factors [3,4,5,6,22].

In Polish literature, there are few articles on eye complications and eye care for ICU patients. In 2018, the Working Group for the Practices of the Polish Society of Anesthetic and Intensive Care Nurses developed and published eye care recommendations for ICU patients [20]. However, there are no studies on the implementation of these recommendations in practice, risk factors for eye complications, and the effectiveness of various methods of eye care among critically ill patients.

Studies in other countries assessing the effectiveness of the use of various eye care algorithms and guidelines have shown the benefits of implementing and systematically applying such protocols in the form of reducing eye surface damage among ICU patients, both in previously published [23,24] and more recent publications [4,25,26,27]. In many studies, various methods of eye care were compared, but the advantage of one method over another was not demonstrated [3,5,28,29,30,31]. Some studies suggest that in the presence of eyelid lagophthalmos, the most effective treatment is applying a polyurethane cover [30,31], but the choice of method should be dictated by the type and degree of eye lesions [3,5]. In our study, several different methods of eye care were used alternately depending on the patient’s condition, such as rinsing with 0.9% NaCl or distilled water, using moisturizing drops and ointments, moisture chambers for patients with lagophthalmos, and ointments with antibiotics in the case of conjunctivitis and keratitis. It was shown that the use of one or more eye care methods according to Polish recommendations was associated with a lower number of ocular complications (rho = 0.590, *p <* 0.001).

This study confirmed the influence of risk factors already known from other reports [3,13], such as the presence of injuries (*p <* 0.01), the use of sedation drugs (*p <* 0.01), mechanical ventilation (*p* < 0.05), and lagophthalmos (*p <* 0.001).

An interesting aspect of the research was showing that the use of a closed suctioning system (CSS) is more beneficial in preventing ocular complications than the use of an open suctioning system (OSS). There are no reports on this subject in the publications known to the authors. In studies comparing CSS and OSS, their impact on various parameters, such as pulmonary volume, desaturation, pain, hemodynamic parameters, and intracranial pressure, was assessed, but their impact on the incidence of ocular complications was not assessed [32,33,34,35,36,37]. According to a literature analysis conducted by Lavigne, the incidence of patient infections and ICU contamination with various microorganisms, including *Escherichia coli*, *Pseudomonas aeruginosa*, *Enterobacter cloacae*, and *Proteus species* is greater in the case of open suction than in the closed method due to the formation of an aerosol during suction [37]. This could potentially also apply to ocular infections, although further studies are required to support this claim. Recommendations based on research results indicate that the suction of secretions from the respiratory tract can be performed using the closed method as well as the open method, with the simultaneous covering of the eyes and placing the ventilator tubes on the side of the bed [4,5,31]. This was also reflected in the recommendations of the Polish Society of Anesthetic and Intensive Care Nurses [20].

The relationship between using sedation and an increased risk of ocular complications, as shown in this study, was also found by other authors [2,16].

It is not entirely clear why incidences of ocular surface lesions were higher among patients who received cardiac drugs (amiodarone, cardiac glycosides, beta-blockers), antibiotics (penicillin, rifampicin, metronidazole), and anticoagulants (rho = 0.424, *p <* 0.001). Studies have shown the presence of toxic neuropathy after amiodarone, but this was true for long-term treatment (several months), and was not associated with damage to the eye’s surface [38,39,40]. Similarly, the relationship between the use of beta-blockers and cataracts has been shown [41,42], but also after longer treatment periods. Both beta-blockers and amiodarone can cause dry eye syndrome and reduce tear production. Anticoagulants can cause subconjunctival and retinal hemorrhages. Cardiac glycosides may also cause conjunctivitis (apart from blurred vision, xantopsia, pupillary disorders, nystagmus, photophopia, and toxic damage to the optic nerve), and rifampicin-conjunctival hyperemia [43,44]. These symptoms were taken into account as signs of ocular complications in this study. It is difficult to say if the ocular complications were the results of using these drugs per se or if they were caused by the overall severe condition of the patients, who required more drugs and had additional risk factors affecting ocular complications; it demands further research.

Our findings confirmed the relationship between the duration of mechanical ventilation and the development of eye complications. Similar results were obtained by Kuruvilla et al. and Mela et al. [27,45]. This is certainly due to the longer exposure to risk factors for eye complications.

Although our study group did not include Coronavirus disease-19 (COVID-19) patients, as the study was conducted before the SARS-CoV-2 (COVID-19) pandemic, it is likely that this group is even more exposed to numerous risk factors for ocular complications such as prolonged mechanical ventilation and hospitalization, non-invasive ventilation, prone positioning [15], and the often severe condition of the patients. Coronavirus disease-19 could also have ophthalmic manifestations [46,47,48].

In our research, a correlation was found between the frequency of ophthalmological consultations and the occurrence of eye complications. Routine, daily examinations by an ophthalmologist are unlikely to be applied in practice, but Mc Hugh et al. [49] have shown high efficacy in detecting ocular complications thanks to the daily assessment of patients’ eyes by trained young physicians who used fluorescein and a pen torch with a blue filter. Appropriate training of doctors and nurses to recognize the early symptoms of eye surface damage and other eye complications seems to be crucial in reducing them. Consulting with an ophthalmologist in the ICU, due to the high risk of patients being exposed to these complications, should be a constant practice in the case of signs of damage to the surface of the eyes, especially in the case of microbial keratitis. This is important considering the consequences of these complications for patients after ICU and hospital discharge. As a result of eye damage, many patients may lose their eyesight and become dependent on others, requiring constant care. Increased awareness of eye complications and their risk factors in ICU settings is therefore crucial to help prevent these complications and maintain the quality of life of patients after discharge.

The limitation of the study is a small sample size and short time of observation. Some correlations reflect only a tendency and the results should not be generalized. The findings could be used to design larger confirmatory research.

## 5. Conclusions

The study confirmed that important risk factors for ocular complications in ICU patients were: injuries, lagophthalmos, mechanical ventilation, length of stay in the ICU setting, and inadequate eye care. Some of the factors can be modified by proper eye care according to protocols. It is necessary to disseminate and precisely follow guidelines and train doctors and nurses in how to make an early diagnosis and treat eye complications in ICU patients.

## Figures and Tables

**Table 1:** Comparison of the occurrence of ocular complications depending on the length of stay of patients in the ICU (Friedman’s ANOVA analysis).

Variable	Day	M	Me	SD	*p*
Occurrence of eye complications (point)	I	2.42	1.00	3.430	*p* < 0.001
	II	2.57	1.00	3.492	
	III	2.67	1.00	3.439	
	IV	3.08	1.00	3.452	
	V	3.37	1.50	3.673	
	VI	4.14	2.00	4.301	
	VII	4.63	2.00	4.752	

**Table 2:** Comparison of the risk of eye complications in men and women (Mann–Whitney U test).

Variable	Gender	N	M	Me	SD	U	Z	*p*
Occurrence of the risk of eye complications	Female	28	21.79	11.00	25.18	655.0	−0.190	0.849
	Male	48	23.52	10.00	24.54			

M—mean; Me—median; SD—standard deviation.

## Data Availability

The data presented in this study are available on reasonable request from the corresponding author.
